# Extent of implementation of food environment policies by the Malaysian Government: gaps and priority recommendations

**DOI:** 10.1017/S1368980018002379

**Published:** 2018-10-02

**Authors:** SeeHoe Ng, Boyd Swinburn, Bridget Kelly, Stefanie Vandevijvere, Heather Yeatman, Mohd Noor Ismail, Tilakavati Karupaiah

**Affiliations:** 1 Early Start, School of Health and Society, University of Wollongong, Wollongong, NSW, Australia; 2 School of Population Health, University of Auckland, Auckland, New Zealand; 3 Faculty of Hospitality, Food and Leisure Management, Taylor’s University, Selangor, Malaysia; 4 Dietetics Program, School of Healthcare Sciences, Faculty of Health Sciences, Universiti Kebangsaan Malaysia, 50300 Kuala Lumpur, Malaysia; 5 School of Biosciences, Faculty of Health and Medical Sciences, Taylor’s University, Selangor, Malaysia

**Keywords:** Food environment, Obesity, Non-communicable diseases, Policy, Upper-middle-income country

## Abstract

**Objective:**

To determine the degree of food environment policies that have been implemented and supported by the Malaysian Government, in comparison to international best practice, and to establish prioritised recommendations for the government based on the identified implementation gaps.

**Design:**

The Healthy Food-Environment Policy Index (Food-EPI) comprises forty-seven indicators of government policy practice. Local evidence of each indicator was compiled from government institutions and verified by related government stakeholders. The extent of implementation of the policies was rated by experts against international best practices. Rating results were used to identify and propose policy actions which were subsequently prioritised by the experts based on ‘importance’ and ‘achievability’ criteria. The policy actions with relatively higher ‘achievability’ and ‘importance’ were set as priority recommendations for government action.

**Setting:**

Malaysia.

**Subjects:**

Twenty-six local experts.

**Results:**

Majority (62 %) of indicators was rated ‘low’ implementation with no indicator rated as either ‘high’ or ‘very little, if any’ in terms of implementation. The top five recommendations were (i) restrict unhealthy food marketing in children’s settings and (ii) on broadcast media; (iii) mandatory nutrition labelling for added sugars; (iv) designation of priority research areas related to obesity prevention and diet-related non-communicable diseases; and (v) introduce energy labelling on menu boards for fast-food outlets.

**Conclusions:**

This first policy study conducted in Malaysia identified a number of gaps in implementation of key policies to promote healthy food environments, compared with international best practices. Study findings could strengthen civil society advocacies for government accountability to create a healthier food environment.

A resolution adopted by the United Nations General Assembly (A/RES/66/2) in 2011 stressed the need for member nations to prevent and control non-communicable diseases (NCD)^(^ ^1^ ^)^. According to the *Sustainable Development Goals Report 2017*, the global progress to reduce the risk of dying from NCD was reported as ‘not sufficiently rapid’ to meet the 2030 target^(^ ^2^ ^)^. This comes in acknowledgement of the global disease burden from NCD which account for 15 million premature deaths annually, with 80 % of this mortality affecting low- and middle-income countries^(^ ^3^ ^)^. Much of this disease burden is diet-related, especially cancers, type 2 diabetes mellitus and CVD, with dietary risk factors contributing to 9·58 % of total disability-adjusted life years^(^ ^4^ ^)^. Notably, high BMI alone explains 5·01 % of disability-adjusted life years from diet-related NCD. Despite the WHO’s goal^(^ ^5^ ^,^ ^6^ ^)^ within the NCD Global Action Plan to halt the rise in overweight and obesity, the current global progress is still far from meeting this goal^(^ ^7^ ^)^. One of the main factors could be industry lobbying^(^ ^8^ ^)^ through public–private partnerships^(^ ^9^ ^)^,which should be better defined to prevent potential risks to achieving NCD goals.

Government policies to support healthy food environments need to be implemented to address dietary risk factors^(^ ^10^ ^–^ ^12^ ^)^ such as high consumption of saturated fat, salt and sugar along with low intakes of whole grains and nuts^(^ ^4^ ^)^ that contribute to diet-related NCD and disability-adjusted life years burden. A healthy food environment enables public access to healthy foods, which is an important determinant for better population food consumption^(^ ^13^ ^)^. Factors contributing to food access include food production, processing, trade and economic policies, marketing and retailing, together with population purchasing power^(^ ^5^ ^,^ ^14^ ^,^ ^15^ ^)^. In this context, availability and affordability of healthier foods, over unhealthy foods, could trigger behavioural changes of individuals^(^ ^15^ ^)^. The ability of governments towards constructive optimal food environment policies requires analyses of their policies against international best practice. However, this type of research is limited globally^(^ ^16^ ^)^ and non-existent in Malaysia.

Malaysia has the highest prevalence of overweight (30·0 % with BMI=25·0–29·9 kg/m^2^) and obese (17·7 % with BMI≥30·0 kg/m^2^)^(^ ^17^ ^)^ adults in the South-East Asian region^(^ ^18^ ^)^. Malaysia is an upper-middle-income and multiracial country with NCD accounting for 73 % of total mortality, of which 20 % are classified as premature^(^ ^19^ ^)^. Ethnic-specific trends of type 2 diabetes mellitus, hypertension and hypercholesterolaemia prevail in Malaysia^(^ ^17^ ^)^, with dietary risks constituting the largest proportion of risks for total disability-adjusted life years (13·4 %) for diet-related NCD^(^ ^4^ ^)^. It is timely therefore, in the light of these alarming public health problems in Malaysia, to independently assess whether the degree of food policies implemented by the government is sound, compared with international best practices that are known to foster healthy food environments. Such study is also highly relevant in the context of the concern and calls made by stakeholders to the Malaysian Government to act now to reduce obesity, by introducing food policies promoting healthy nutrition^(^ ^20^ ^)^.

The Healthy Food-Environment Policy Index (Food-EPI) was developed by the International Network for Food and Obesity/NCDs Research, Monitoring and Action Support (INFORMAS)^(^ ^13^ ^)^ to assess the level and range of policy actions by national-level governments. It has been used in Australia, New Zealand, UK and Thailand^(^ ^16^ ^,^ ^21^ ^–^ ^24^ ^)^. Notably, researchers in New Zealand repeated the evaluation in 2017 after the baseline was first conducted in 2014 and observed progress for some indicators of policy actions^(^ ^24^ ^)^. The tool has been identified by Phulkerd *et al*.^(^ ^25^ ^)^ as one of the three ‘high’ quality tools and processes to evaluate national food environment policy implementation in a recent review of tools. Given the experience of Food-EPI application in these Asia-Pacific countries, we collaborated with the INFORMAS in using this tool for evaluating Malaysia’s food environment policies. Our aims were to: (i) determine the degree of implementation of food environment policies and supports provided by the Malaysian Government, against international best practices; and (ii) establish prioritised recommendations for the government based on the identified implementation gaps.

It is envisaged that the study outcomes will contribute (i) a baseline reference for future policy formulations in Malaysia and (ii) towards nurturing collaborations in combating obesity and NCD in the South-East Asian countries. One example of this is the Bandar Seri Begawan Declaration, which was adopted at the 23rd Association of Southeast Asian Nations (ASEAN) Summit in Brunei Darussalam. The Declaration prioritised actions to develop a framework within ASEAN Member States for unhealthy foods and beverages as one of the means for combating obesity and NCD^(^ ^26^ ^)^. Member states are committed to conduct national analyses of the food environment policies to identify implementation gaps. This would eventually lead towards a uniform action to tackle diet-related NCD within the ASEAN framework and creating opportunities for food-related trade targeting a healthy food environment.

## Methods

### Background information on Food-EPI

**Fig. 1:**
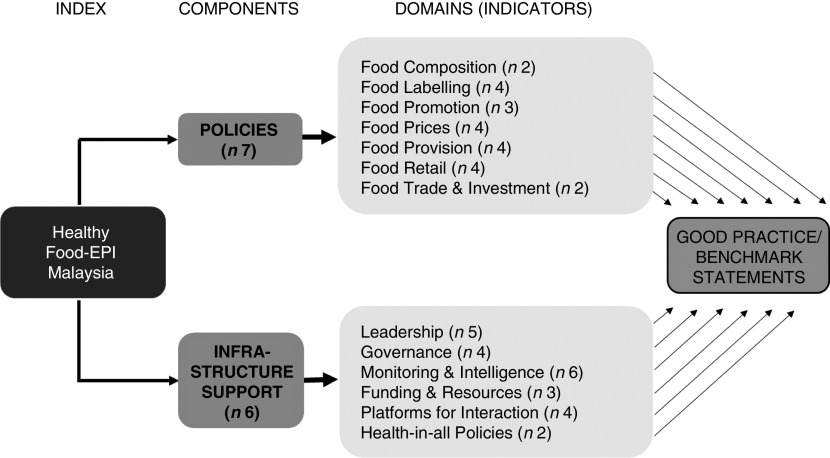
Components and domains of the Healthy Food-Environment Policy Index (Food-EPI) tool (adapted from Swinburn *et al*.^(^ ^13^ ^)^)

The Food-EPI tool comprises two components, ‘Policy’ and ‘Infrastructure Support’, spanning thirteen domains (e.g. food labelling, food promotion, leadership, governance, etc.) composed of forty-seven indicators as described in Fig. 1. The tool was developed by Swinburn *et al*.^(^ ^13^ ^)^ through a consultation process with international food policy experts, in which good practice statements were formulated based on the review of policy documents and the experts’ opinions.

### Adaptation of the Food-EPI tool

The Food-EPI tool and process developed by Swinburn *et al*.^(^ ^13^ ^)^ was adapted to include local context. An in-depth discussion on the suitability of the tool was conducted on 15–16 August 2016 in Kuala Lumpur, Malaysia. This discussion was attended by five INFORMAS members with three Food-EPI researchers from Malaysia and the remaining from Singapore, Vietnam and Thailand. Food-EPI indicators used in countries such as New Zealand^(^ ^16^ ^)^ and Australia^(^ ^22^ ^)^ were discussed and complemented by a sharing session from the Thai Food-EPI researchers. In comparison to the Food-EPI indicators used in New Zealand^(^ ^16^ ^)^ and Thailand^(^ ^21^ ^)^, the Food-EPI tool adapted for Malaysia includes indicators specific to: (i) food composition policy targeting foods away from home and processed foods; and (ii) food promotion policy through broadcast and non-broadcast media. Indicators were also expanded for other two domains, including: (i) food retail policy at food-service outlets (e.g. hawkers); and (ii) commitments to funding and resources for the Malaysian Health Promotion Board. In total, the Food-EPI Malaysia spanned thirteen domains with forty-seven indicators.

### Food-EPI process

**Fig. 2:**
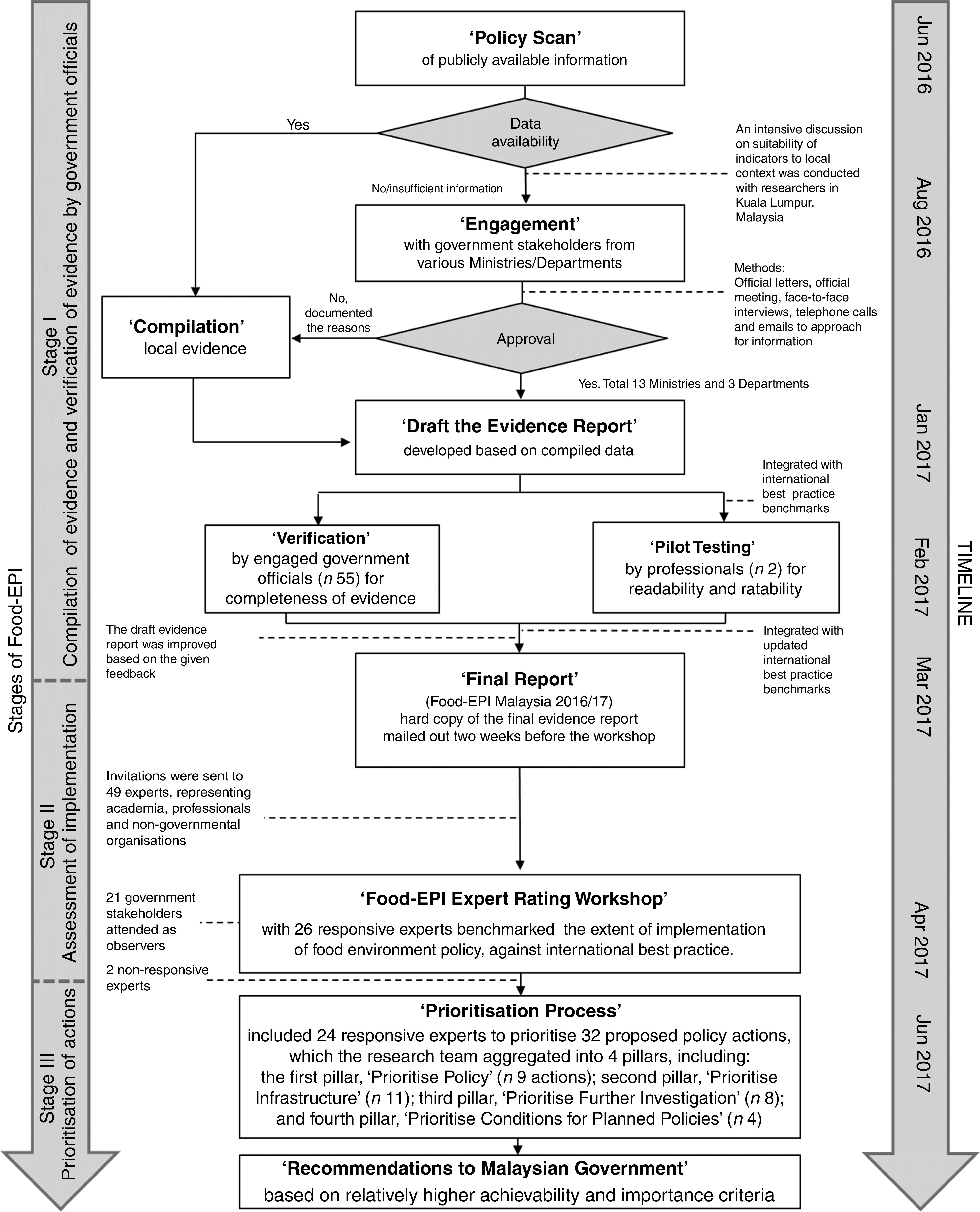
The Healthy Food-Environment Policy Index (Food-EPI) process in Malaysia, 2016/17

Figure 2 shows the three main stages involved in the Food-EPI process according to the INFORMAS protocol. The study was conducted in the English language as the English proficiency level of government officers was above average. The study received approval from the Research Ethics Committee, The National University of Malaysia (UKMPP1/111/8/JEP-2016-394); the Social Science Human Research Ethics Committee of the University of Wollongong (HE16/297); and the Medical Research and Ethics Committee, Ministry of Health Malaysia (NMRR-17-195-34142(IIR)).

#### Stage I: Compilation of evidence and verification of evidence by government officials

Stage I involved a ‘Policy Scan’ of publicly available information on the implementation of forty-seven food environment policy and infrastructure support indicators. Local evidence of implementation was contributed by thirteen Ministries and three Departments (see online supplementary material, Supplemental Table 1). The ‘Engagement’ process involved official letters, meetings, face-to-face interviews, telephone calls and emails. Data for each indicator were collected based on the degree of implementation by the government. The scope included actions and policies implemented by the government at federal level, government-funded actions undertaken by non-governmental organisations (NGO) as well as the federal government’s intentions and plans to develop or implement policies in the near future. The data collection and ‘Compilation’ took 10 months (June 2016 to March 2017) and an initial draft of the evidence was verified by government stakeholders (*n* 55).

For the purposes of ‘Pilot Testing’, the draft evidence report was integrated with the international best practice benchmarks. Two non-governmental professionals, one with nutrition training and one without, pilot-tested the draft evidence report for readability. They also assessed ease of rating for each indicator based on 4-point Likert scales (1=‘difficult’; 2=‘fairly difficult’; 3=‘fairly easy’; 4=‘easy’). Most indicators were perceived to be easy to rate, except three, which were improved based on constructive feedback.

The ‘Final Report’ (Food-EPI Malaysia 2016/17) included verified evidence from the government stakeholders, together with the revised international best practice exemplars (‘benchmarks’) to be used later by the experts to perform the ratings for each indicator. The benchmarks were updated from the NOURISHING framework database of the World Cancer Research Fund^(^ ^27^ ^)^. They also incorporated recommendations by the international experts on food, nutrition and obesity whose knowledge was current up to 15 March 2017. An example of an international best practice benchmark for the menu labelling indicator is the Special Act on Safety Control of Children’s Dietary Life in South Korea. It requires all chain restaurants with ≥100 establishments to display nutrient information on menus including energy, total sugars, protein, saturated fat and sodium content^(^ ^28^ ^)^.

#### Stage II: Assessment of implementation

In total, forty-nine multi-sector experts from universities (*n* 25), NGO or non-profit organisations (*n* 21) and professionals (*n* 3) were approached through official invitation letters. Each invitation included an information sheet and consent form. Invitations were followed up with emails and/or telephone calls to obtain the written informed consent and declaration of conflicts of interest prior to the rating process. Ten invitees declined to participate, followed by eight last-minute withdrawals and four non-responsive invitees. One invitee was initially invited as an academic but was reassigned to be an NGO representative. Two weeks prior to the ‘Food-EPI Expert Rating Workshop’, the Food-EPI Malaysia 2016/17 evidence report was disseminated to all participating experts for preparative reading. In all, twenty-four experts provided the rating through the workshop, while two experts responded via email. Additionally, government stakeholders attended the workshop as observers.

Upon registration at the workshop, each expert received a personalised rating form and rating device with non-identifiable code. The experts were required to first fill in their demographic data such as age, gender, ethnicity, professional background and years of working experience on the rating form. Information of expertise was obtained via a short answer question. Before a rating for an indicator was conducted, the experts were briefed about global best practice benchmarks for comparison with local data. Clarifications of details were allowed and this discussion facilitated the experts forming a judgement on the ‘quality’ of government policies and the extent of their implementation. The rating for each indicator was conducted using an Interactive Voting System (IVS-Basic program, Netherlands), an automated audience response tool to ensure anonymity. A 10-point Likert scale was used (1=‘low implementation’ (0–10 %) to 10=‘high implementation’ (90–100 %), compared with best practice). The experts were also provided rating forms to manually record scores and comments, which were incorporated into data analysis.

The Interactive Voting System facilitated the experts to rate each indicator, generated the rating results live, and allowed subsequent voting to propose an action to the government or not. Prior to the rating, discussions between researchers, stakeholders and experts clarified any issues relating to the specific indicators. For all voted indicators (*n* 47), actions were proposed when a two-thirds majority of experts (≥66 %) voted ‘yes’. Actions were decided based on one of three criteria, specifically when there was: (i) poor implementation compared with international best practice; (ii) a need to broaden the scope of the current plans of government; or (iii) more evidence required to support action. Proposed policy actions were discussed and shortlisted. Post-workshop emails were sent to government stakeholders who attended as observers, and seven out of fifteen provided constructive feedback to fine-tune the wording of the proposed actions.

#### Stage III: Prioritisation of actions

After the workshop, the experts prioritised the proposed actions according to ‘importance’ and ‘achievability’ criteria. ‘Importance’ of a proposed policy action took account of the relative need (size of the implementation gap), impact (effectiveness of the action such as the reach and effect size), effects on equity (effects on reducing diet-related health inequalities), and any other positive and negative effects of the action. In terms of the ‘achievability’, feasibility (level of easiness to be implemented), acceptability (level of support from key stakeholders), affordability (implementation cost) and efficiency (cost-effectiveness) of that proposed policy were taken into account^(^ ^28^ ^)^. No criterion was measured in the context of time frame.

The shortlisted proposed actions were aggregated by the researchers into four pillars according to the nature of the actions. The first pillar, ‘Prioritise Policy’, summarised actions under the ‘Policy’ component, while the second pillar, ‘Prioritise Infrastructure’, included actions under the ‘Infrastructure Support’ component. The third pillar, ‘Prioritise Further Investigation’, covered actions in areas of the food environment where local data were lacking and there was complexity associated with implementation, which required further investigation before introduction could be justified. The fourth pillar, ‘Prioritise Conditions for Planned Policies’, included actions that were in line with the intentions or national plans of the government, but consensus and prioritisation from the experts was required to broaden the scope or areas. An Excel file comprising an instruction manual (with video tutorial), a summary of the rating results and four spreadsheets of proposed policy actions arranged based on the pillars was emailed to the experts for the ‘Prioritisation Process’.

Actions within each pillar were allocated with an initial 5 points for each criterion. Maximum points were set for each pillar based on the number of proposed actions. For example, the first pillar ‘Prioritise Policy’ was set at 45 maximum points, in each of the ‘importance’ and ‘achievability’ columns (i.e. 9 proposed actions×5 points=45 maximum points for each of ‘importance’ and ‘achievability’ columns). The experts then reallocated points (minimum value=0) for each proposed action, within the maximum points set for each pillar, in each of the ‘importance’ and ‘achievability’ column. In addition, the experts could provide comment(s) on the proposed actions, if any. Proposed actions with relatively higher ‘achievability’ and ‘importance’ based on the points allocated by the experts were packaged into the ‘Recommendations to Malaysian Government’.

### Data analysis

The statistical analysis was conducted using the statistical software package IBM SPSS Statistics version 21.0. Ratings of indicators by the experts were calculated based on the scoring from the rating forms after the workshop and expressed as mean percentages of implementation compared with best practice. The mean rating for each indicator was subsequently categorised into ‘very little, if any’ (<25 %), ‘low’ (26–50 %), ‘medium’ (51–75 %) and ‘high’ (>75 %) implementation, compared with best practice. Inter-rater reliability (Gwet AC2 coefficient) was calculated using AgreeStat software version 2013·1 (Advanced Analytics, Gaithersburg, MD, USA). Rater sample or fraction response rate was fixed as 53·06 %, while subject sample fraction was fixed as 100 % based on all Food-EPI indicators being rated.

Differences in ratings based on the experts’ professional background, academia/professional *v*. NGO, were tested. For each pillar, average points on ‘importance’ and ‘achievability’ scales for each proposed action were mapped using a four-quadrant scatter graph. Top recommendations were developed from points allocated by the experts providing subjective opinions based on ‘achievability’ and ‘importance’ criteria. The higher the points allocated for the ‘achievability’ and ‘importance’ criteria, the more likely the proposed policy actions to be assigned at the upper-right quadrant of the scatter graph, indicating the top recommendations. Since the total ratings and allocated prioritisation points by the experts did not fulfil normality assumption, Mann–Whitney *U* tests were performed. Statistical significance was set as *P* value threshold of 0·05 for all data analysis.

## Results

### Characteristics of the experts

Twenty-six experts participated in Stage II of the Food-EPI process, with a response rate of 53 %. The mean age of the experts was 49·4 (sd 11·2) years with equal gender distribution (male/female=13/13). Most of the experts were from non-governmental/non-profit organisations (*n* 15; Table 1) and twenty-one out of thirty-three invited government stakeholders attended the rating workshop as observers (online supplementary material, Supplemental Table 1).

**Table 1:** Profile of experts (*n* 26) participating in the Healthy Food-Environment Policy Index (Food-EPI) process in Malaysia, 2016/17

Characteristic	*n*	%
Age (years)		
21–30	1	3·8
31–40	6	23·1
41–50	5	19·2
51–60	9	34·6
61 or above	5	19·2
Gender		
Male	13	50·0
Female	13	50·0
Ethnicity		
Malay	13	50·0
Chinese	5	19·2
Indian and others	8	30·8
Professional background		
Academia/professionals	11	42·3
Non-government/non-profit organisation	15	57·7
Working experience (years)		
0–10	4	15·4
11–20	10	38·5
21–30	11	42·3
31–40	1	3·8
Expertise*		
Agriculture	1	3·8
Consumerism	3	11·5
Nutrition and dietetics	10	38·5
Economics (health economics, macroeconomics analysis & agribusiness)	4	15·4
Public health and health promotion	6	23·1
Medical specialists	4	15·4
Diet-related non-communicable diseases	8	30·8
Psychology (eating & consumer behaviour)	2	7·7
Obesity	4	15·4

* More than one field of expertise may be stated in the consent form. Hence, the total is not equivalent to twenty-six experts.

### Extent of policy implementation compared with international best practice

**Fig. 3:**
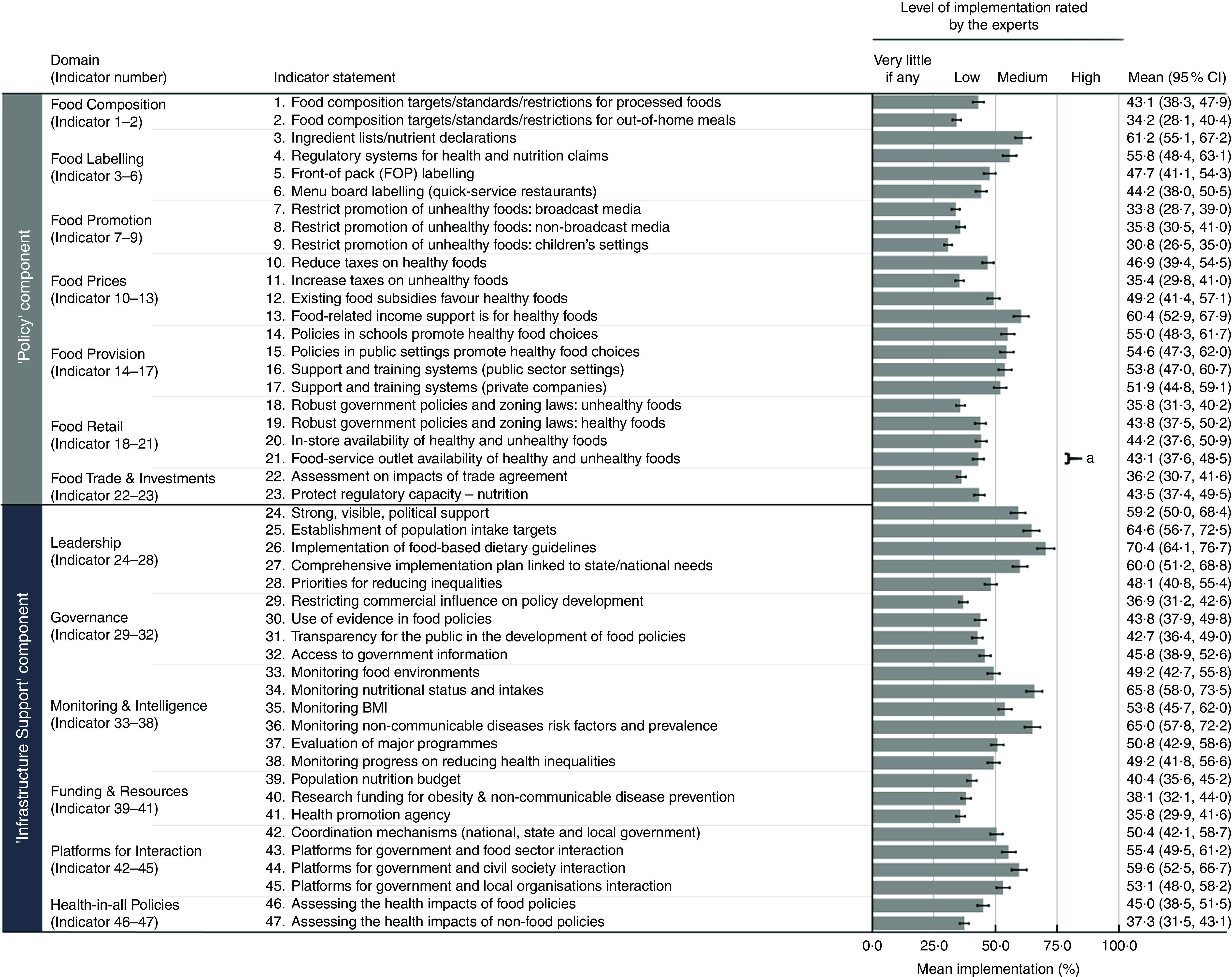
Mean percentage of implementation (■), with their SE represented by error bars, for indicators under ‘Policy’ and ‘Infrastructure Support’ components as rated by experts (*n* 26) participating in the Healthy Food-Environment Policy Index (Food-EPI) process in Malaysia, 2016/17. Note: The 95 % CI for the mean value is also provided for each indicator. ^a^Significant difference in experts’ percentage ratings between academia/professional (*n* 11) and non-governmental/non-profit organisations (*n* 15): *P*<0·05 (Mann–Whitney *U* test)

Inter-rater reliability of ratings performed by the experts was 0·65 (95 % CI 0·56, 0·74). Nearly two-thirds of the indicators (62 %) were rated as ‘low’ implementation, followed by 38 % as ‘medium’ implementation, while no indicator was rated at either ‘very little, if any’ or ‘high’ implementation (Fig. 3). Within the ‘Policy’ component of the Food-EPI, only the ‘food provision’ domain (in relation to schools, public settings and private companies) was rated as ‘medium’ implementation for all indicators (Food Provision, Indicators 14–17). The top three indicators with the highest ratings (‘medium’ implementation) within the policy component were: (i) ingredient lists and nutrient declarations (Food Labelling, Indicator 3: 61·2 %); (ii) food-related income support for healthy foods (Food Prices, Indicator 13: 60·4 %); and (iii) food regulatory systems for health and nutrition claims (Food Labelling, Indicator 4: 55·8 %). The three indicators that received the lowest ratings were: (i) restricting the exposure and power of unhealthy food promotions in children’s settings (Food Promotion, Indicator 9: 30·8 %) and (ii) through broadcast media such as television (Food Promotion, Indicator 7: 33·8 %); and (iii) food composition standards for out-of-home meals (Food Composition, Indicator 2: 34·2 %).

More indicators under the ‘Infrastructure Support’ component were rated as ‘medium’ implementation in comparison with the ‘Policy’ component (11/24 *v*. 7/23, respectively). The top three indicators with the highest ratings for infrastructure support were: (i) the establishment and implementation of food-based dietary guidelines (Leadership, Indicator 26: 70·4 %); (ii) monitoring of population nutritional status and intakes against targets (Monitoring and Intelligence, Indicator 34: 65·8 %); and (iii) monitoring of NCD risk factors and prevalence (Monitoring and Intelligence, Indicator 36: 65·0 %). The lowest ratings were for: (i) having a funding stream for a statutory health promotion agency (Funding and Resources, Indicator 41: 35·8 %); (ii) restricting commercial influence in policy development (Governance, Indicator 29: 36·9 %); and (iii) having processes to assess health impacts during the development of non-food policies (Health-in-all Policies, Indicator 47: 37·3 %).

Across both components, only one indicator indicated a significant difference in score according to the experts’ professional background. Academia rated significantly higher implementation of policy encouraging increased access to healthy foods and limiting access to unhealthy foods through regulating food-service outlets (Food Retail, Indicator 21) than NGO (48·2 (sd 9·8) % academia *v.* 39·3 (sd 14·9) % NGO; *U*=44·0, *P*=0·036). However, both ratings were classified as ‘low’ implementation.

### Prioritisation of actions

In total, thirty-two proposed policy actions were shortlisted across forty-seven indicators for the post-workshop ‘Stage III: Prioritisation of actions’. These were categorised into pillars with nine actions under the first pillar, ‘Prioritise Policy’; eleven under the second pillar, ‘Prioritise Infrastructure’; eight under the third pillar, ‘Prioritise Further Investigation’; and four under the fourth pillar, ‘Prioritise Conditions for Planned Policies’ (see online supplementary material, Supplemental Table 2). There were twenty-four responsive experts of the twenty-six experts who performed the ‘Prioritisation Process’.

**Fig. 4:**
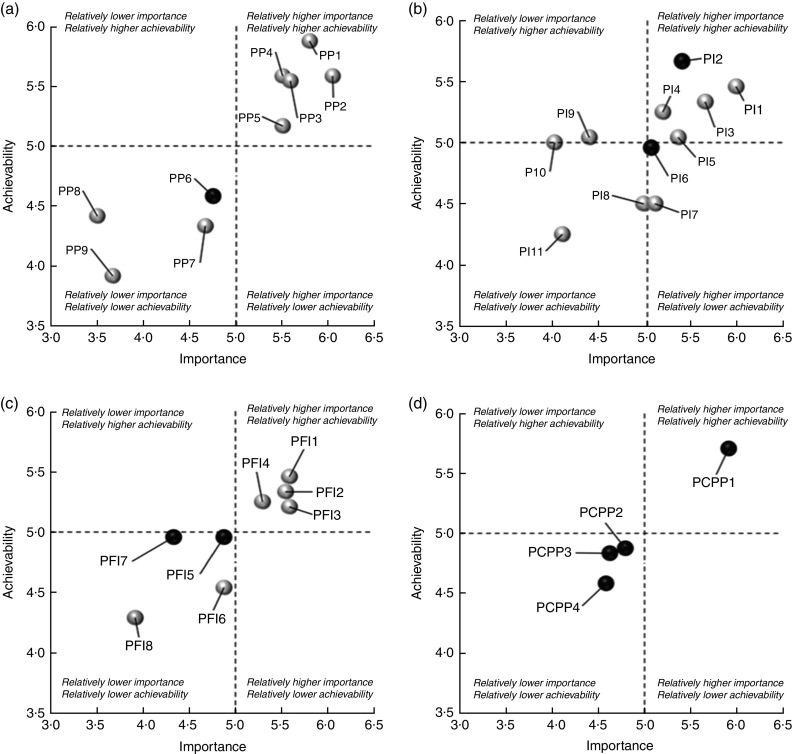
Scatter plots for each pillar based on ‘importance’ and ‘achievability’ criteria as rated by experts (*n* 24) participating in the Healthy Food-Environment Policy Index (Food-EPI) process in Malaysia, 2016/17. (a) First pillar, ‘Prioritise Policy’ (PP). PP1=Enact a policy to restrict unhealthy marketing in children’s settings. PP2=Create regulations to restrict unhealthy broadcast promotions to children. PP3=Display energy menu board labelling in fast-food outlets and other food outlets. PP4=Set sodium targets for selected food groups. PP5=Introduce sugary drink taxes. (b) Second pillar, ‘Prioritise Infrastructure’ (PI). PI1=Designate research funding for obesity and diet-related non-communicable disease reduction; PI2=Optimise existing system and strengthen referral mechanism. PI3=Increase funding for population nutrition promotion commensurate to the unhealthy diet burden. PI4=Strengthen access to information related to public consultation. PI5=Strengthen sustainable funding and function of the Health Promotion Board. (c) Third pillar, ‘Prioritise Further Investigation’ (PFI). PFI1=Investigate opening hours of fast-food restaurants and their placement of outlets near schools and residential areas. PFI2=Investigate restriction on unhealthy non-broadcast marketing to children. PFI3=Investigate food composition standards for added sugar and saturated fats. PFI4=Investigate price rises in fruit and vegetables and potential fiscal policies. (d) Fourth pillar, ‘Prioritise Conditions for Planned Policies’ (PCPP). PCPP1=Implement planned regulations (i.e. sodium and total sugar labelling and quantitative ingredient declarations) and broaden the declaration to added sugars. Notes: (i) Only summary statements of proposed policy actions with relatively higher ‘importance’ and ‘achievability’ (upper-right quadrant of Fig. 4(a)–(d)) are stated above; for further details, please refer to the online supplementary material, Supplemental Table 2. (ii) For further details of the proposed policy actions appearing in other quadrants of Fig. 4(a)–(d), please refer to Supplemental Table 2. (iii) Both axes do not start from ‘0’ to give a better illustration of the distribution for proposed policy actions as per pillar. (iv) Dark/black bubbles refer to indicators with ‘medium’ implementation rated by the experts, while white/grey bubbles refer to ‘low’ implementation, against international best practice benchmarks

The top fifteen recommendations with relatively higher ‘achievability’ and ‘importance’ (Fig. 4(a)–(d)) were formulated into an action package, which comprised eight corresponding domains such as ‘Food Promotion’, ‘Food Labelling’, ‘Food Composition’, ‘Food Retails’, ‘Food Prices’, ‘Funding and Resources’, ‘Monitoring and Intelligence’ and ‘Governance’. The consensus for these recommendations did not differ significantly by the experts’ professional background (*P*>0·05). Under the ‘Policy’ component, the experts prioritised recommendations to: (i) enact a policy to restrict unhealthy food and beverage marketing in children’s settings (Prioritise Policy 1) and media (Prioritise Policy 2 and Prioritise Further Investigation 2); (ii) continue to implement planned regulations on mandatory nutrition labelling and broaden the scope to include added sugars declaration on packaged foods (Prioritise Conditions for Planned Policies 1), as well as display energy labelling on menu boards for fast-food chains (Prioritise Policy 3); (iii) establish sodium targets (Prioritise Policy 4) and investigate food composition standards for added sugar and saturated fat (Prioritise Further Investigation 3); (iv) investigate restriction on opening hours of fast-food restaurants and seek opportunities to restrict the new establishment near schools and residential areas (Prioritise Further Investigation 1); and (v) introduce taxes on sugary drinks with revenues applied to healthy diets for children (Prioritise Policy 5) and investigate price rise in fruits and vegetables (Prioritise Further Investigation 4).

Recommendations pertaining to infrastructure support within the action package included to: (i) continuously designate funding for research and ensure population nutrition promotion budget to be commensurate with the size of the health burden from unhealthy diets as well as to strengthen sustainable funding for the Malaysian Health Promotion Board (Prioritise Infrastructure 1, 3 and 5); (ii) strengthen access to information related to public consultation and provide open access for submissions by the main affected parties (Prioritise Infrastructure 4); and (iii) optimise usage of existing monitoring (e.g. National Physical Fitness Standard – anthropometric measurements for children aged 10–17 years old) and provide appropriate feedback and referral mechanism (Prioritise Infrastructure 2).

## Discussion

Overall, the experts’ views concurred on the need for improvement in the Malaysian Government’s implementation of food environment policies as well as the required infrastructure to support implementation. This view was reflected in the fact that none of the indicators was scored as ‘high’ implementation (0/47). However, medium ratings were ascribed to: (i) food labelling, particularly regulatory systems for nutrient declarations and nutrition claims; (ii) institutional food provision guidelines or standards; (iii) policy leadership; (iv) monitoring and intelligence for nutritional status and intake, prevalence and risk factors of NCD; and (v) platforms for interaction between government and the food sector, civil society or local organisations. The findings indicated that Malaysia was not meeting any of the recognised international benchmarks.

Using the same Food-EPI tool, ‘high’ implementation has been reported for some indicators in Asia-Pacific countries such as New Zealand (7/47) and Thailand (5/30)^(^ ^21^ ^,^ ^24^ ^)^. Similar to Malaysia, the UK has also been rated as not achieving ‘high’ implementation for any indicator (0/48)^(^ ^23^ ^)^, even though the UK policies for traffic light front-of-pack labelling and mandatory nutritional standards for school foods have been recognised as international benchmarks by the INFORMAS^(^ ^28^ ^)^. The lower rating by the UK experts would likely relate to the incomplete implementation of these policies. Therefore, in applying the Food-EPI tool across countries, this would likely be subject to local experts’ experience and knowledge of the policy situation. This may hinder cross-country comparisons in absolute ratings but would still provide useful comparisons of relative priorities and performance across indicators. Civil interest has been identified as a major factor influencing experts’ policy ratings^(^ ^21^ ^)^. Disparities that could affect ratings are experts’ profile, experts’ championing of consumer interests, experts’ health perceptions towards disease and their perceptions about government’s role in policy implementation. However, a main strength of the Food-EPI is that it allows monitoring policy implementation over time within countries and a ranking of the relative priority areas across countries.

Among seven domains under the ‘Policy’ component, ‘Food Promotion’ was the only domain with all three proposed policy actions being prioritised by the experts for the top fifteen recommendations. The experts rated the existing self-regulatory approaches^(^ ^29^ ^)^ (Responsible Advertising to Children – Malaysia Pledge; Guideline for Advertising of Fast Food) under the food promotion indicator relating to the restriction on unhealthy food marketing on broadcast media as relatively weak policies. This was in comparison with the legislative approaches in Chile (Law No. 20.606 of the Law of Nutritional Composition of Food and Advertising) and South Korea (Article 10 of the Special Act on the Safety Management of Children’s Dietary Life)^(^ ^30^ ^)^. This ‘low’ rating in Malaysia assigned to responsibility towards children’s advertising might relate to the four times higher rate of unhealthy food advertising to healthy food advertising found on normal days. The comparative rate changed to nine times during school holidays, and specifically increased to ten times for the peak viewing time^(^ ^31^ ^)^. Kraak *et al*.^(^ ^32^ ^)^ reported that despite multiple countries endorsing restricted unhealthy food marketing to children, the progress by most governments has not been robust. Therefore, the Malaysian Food-EPI experts, in sharing similar concerns about unhealthy food advertising, prioritised an action to restrict unhealthy food marketing to children, as also has been recommended by New Zealand, Thailand, Australia and the UK^(^ ^16^ ^,^ ^21^ ^–^ ^24^ ^)^.

The experts supported regulation plans by government for the mandatory declaration of total sugar and sodium of packaged foods^(^ ^33^ ^)^ and further prioritised an action to include ‘added sugar’ content information on food labels. Another priority they identified was the need to display energy content of foods in fast-food outlets. Such action has borne benefits elsewhere; for example, policy changes in New York^(^ ^34^ ^)^ and New South Wales, Australia^(^ ^35^ ^)^ resulted in lower-energy food purchases. As Bruemmer *et al*.^(^ ^36^ ^)^ suggested, energy declaration would stimulate menu reformulation for less-energy-dense foods with lower content of nutrients of concern. Experts also advocated for establishing specific sodium targets for selected food groups. This recommendation aligns with the Argentinian Law on Maximum Levels of Sodium Consumption 2013, which established gradual sodium reduction targets for selected processed foods^(^ ^37^ ^)^.

Poor funding has previously been identified as a factor contributing to lack of government action to foster healthy food policies in low- and middle-income countries^(^ ^38^ ^)^, as is Malaysia. Anderson *et al*.^(^ ^39^ ^)^ also highlighted funding issues and recommended that effective budget requests should project health expenditure as an investment plan, rather than merely a cost. Funding was identified as an issue in the current study, with the experts suggesting the introduction of taxes on sugary drinks might increase revenue for government. The Mexican experience post-implementation of the sugar tax indicated a 9·7 % reduction in sugary drink purchases, mostly at the lowest socio-economic level and without increasing unemployment rates^(^ ^40^ ^,^ ^41^ ^)^. Two ASEAN countries, Brunei^(^ ^42^ ^)^ and Thailand^(^ ^43^ ^)^, also enacted a sugar tax on beverages with high sugar content in 2017.

Within the South-East Asia region, only Thailand^(^ ^21^ ^)^ and Malaysia have conducted a Food-EPI assessment. Notably, the top priority recommendations in Thailand (*n* 11) and Malaysia (*n* 15) shared some similarities, such as the recommendations related to the protection of marketing to children, particularly in children’s settings. In terms of food composition, both countries considered the necessity to set saturated fat, sugar and sodium levels in major food groups. Food labelling was another domain, highlighting the need to display nutrients of concern such as sugar and salt contents on the nutrition information panel. In relation to monitoring population trends in body weight, both countries emphasised the need to optimise the use of existing data (e.g. fitness, anthropometric measurements) with appropriate feedback mechanisms and follow-up actions. Notably, some recommendations in Malaysia might be more impactful such as the support for ‘added sugars’ in the nutrient label and a regulatory approach to restrict the exposure and power of broadcast promotions for unhealthy foods and beverages to children. Overall, this implies that there is a great opportunity for countries in the same region to work together on joint priorities to maximise a greater capacity.

The strength of the present study was the use of the established tool, coupled with a broad and active engagement with relevant stakeholders across ministries, who facilitated retrieval of comprehensive policy evidence. This evidence transformed a qualitative comparison of policy into a measurable quality scoring of the implementation by the local non-government expert evaluation. These findings provide baseline benchmarks for the Malaysian Government and might be applicable to be key performance indices for the relevant leading ministries. Surveillance of current and future policies will be possible using the accountability criteria outlined herein, as reported in New Zealand when the Food-EPI was used 3 years after the first assessment in 2014^(^ ^24^ ^)^. In addition, civil societies, in their role as ‘society’s conscience’ holding governments to account^(^ ^44^ ^)^, could adopt the actions prioritised in the present study for their advocacy goals, creating windows of opportunity for policy change.

Some limitations are noted when interpreting our findings. Full access to government data considered ‘highly confidential’ (e.g. funding and resources for research) was limited to the researchers as free access to such information is not obligatory^(^ ^45^ ^)^. However, this obstacle was overcome through a systematic approach of compiling projects or information that was publicly available, followed by official requests of the relevant data from respective stakeholders. Additionally, invitees who declined to participate cited reasons such as personal or work commitments, lack of expertise in this area of research or even disappointment over previous engagements with policy stakeholders. Although only twenty-six experts participated in the rating process, their long years of work experience (mean 20·4 (sd 9·1) years) and diverse scope of expertise have contributed adequately to this robust policy rating process.

The present study is the first to benchmark the degree of implementation of food environment policies of the Malaysian Government, against international best practice by the INFORMAS. The study provides an outcome-oriented approach to policy evaluation through a rating process performed by independent local experts from academia, professionals and NGO to the Malaysian Government. The new knowledge generated by this assessment will become a reference for future agendas in developing a healthy food environment for Malaysia. It is also expected that study outcomes will contribute towards nurturing regional collaborations, particularly through the ASEAN platform, to combat obesity and NCD.

## Supplementary material

For supplementary material accompanying this paper visit https://doi.org/10.1017/S1368980018002379. click here to view supplementary material
