# Supplementary material for: Extent of implementation of food environment policies by the Malaysian Government: gaps and priority recommendations
Source: Public Health Nutr. 2018 Oct 2;21(18):3395–406. doi: 10.1017/S1368980018002379 (PMC10260876; doi:10.1017/S1368980018002379)
Supplement: Supplementary file 1 [file S1368980018002379sup001.docx]

**Supplementary Table 1 List of Engaged Government Stakeholders**

| No | Ministry | Data Collection and Verification | Attended Rating Workshop |
| --- | --- | --- | --- |
|  | Ministry of Health | √ | √ |
|  | Ministry of Agriculture and Agro-based Industry | √ | √ |
|  | Ministry of Communications and Multimedia Malaysia | √ | √ |
|  | Ministry of Education | √ | √ |
|  | Ministry of Higher Education^a^ | √ | X |
|  | Ministry of International Trade and Industry | √ | √ |
|  | Ministry of Domestic Trade, Co-operatives and Consumerism | √ | √ |
|  | Ministry of Plantations Industries and Commodities | √ | √ |
|  | Ministry of Science, Technology and Innovation^a^ | √ | X |
|  | Ministry of Urban Wellbeing, Housing and Local Government | √ | √ |
|  | Ministry of Rural and Regional | √ | √ |
|  | Ministry of Women, Family and Community Development | √ | √ |
|  | Ministry of Defence | √ | √ |
|  | Prime Minister’s Department | √ | √ |
|  | Department of Statistics Malaysia^a^ | √ | X |
|  | Royal Malaysian Customs Department^a^ | √ | X |

^a^Government stakeholders only involved in data contribution and verification for indicators related to research funds and food prices (in particular consumer price index and taxes).

**Supplementary Table 2 Proposed Policy Actions for Prioritisation**

| Pillar | Pillar Code | Domain  [Corresponding indicator(s)] | Average Aggregated ratings | Proposed Policy Action | Prioritisation Point  (%overall) | Rank based on pillar | Overall Rank |
| --- | --- | --- | --- | --- | --- | --- | --- |
| First Pillar – Prioritise Policy" (PP) | PP1 | Food Promotion (Indicator 9) | 30.8 | The government should enact a policy to restrict unhealthy food and beverage marketing (including sponsored education, sports and cultural activities) in children’s settings. | 280 (3.6) | 1 | 1 |
|  | PP2 | Food Promotion (Indicator 7) | 33.8 | The government should create regulations to restrict the exposure and power of broadcast promotions for unhealthy food and beverages to children. | 279 (3.6) | 2 | 2* |
|  | PP3 | Food Labelling (Indicator 6) | 44.2 | The government should require all chain fast food outlets (>20 outlets nationally) to display calorie labelling on menu boards and promotes their use in other food outlets (e.g. *mamak* stalls). | 267 (3.5) | 3 | 5 |
|  | PP4 | Food Composition (Indicator 1) | 43.1 | The government should set sodium targets for selected food groups. | 266 (3.5) | 4 | 6 |
|  | PP5 | Food Prices  (Indicator 11) | 35.4 | The government should introduce taxes on sugary drinks with the funding raised applied to promoting healthy diets for children. | 256 (3.3) | 5 | 12 |
|  | PP6 | Food Labelling (Indicator 4) | 55.8 | The government should create an additional nutrient profiling criterion making nutrient claims to ensure unhealthy foods high in fat, sugar and salt are not permitted to make nutrient claims. | 224 (2.9) | 6 | 24 |
|  | PP7 | Food Labelling (Indicator 5) | 47.7 | The government should set robust criteria to be implemented in stages for nutrients of concern for interpretive front of pack label systems for processed foods (including those manufactured by small and medium enterprises (SMEs). | 216 (2.8) | 7 | 27 |
|  | PP8 | Food Retail  (Indicator 21) | 43.1 | The government should strengthen the nutrition components of the BeSS programme and considers providing reductions in renewal of license fees for active participants. | 190 (2.5) | 8 | 31 |
|  | PP9 | Food Trade & Investments  (Indicator 22) | 36.2 | The government should identify opportunities to strengthen the health impact component (specifically nutrition) of the National Impact Assessment, improve domestic regulations by applying nutrition standards or through health certification control the import of non-nutritious foods in order to minimize the negative impact of trade agreements on population nutrition and health. | 182 (2.4) | 9 | 32 |
| Second Pillar – "Prioritise Infrastructure" (PI) | PI1 | Funding & Resources  (Indicator 40) | 38.1 | The government should continue to designate the reduction in obesity and diet-related NCDs and their inequalities as a priority area for research in a coordinated way across its research funding mechanisms by different agencies. | 274 (3.6) | 1 | 4 |
|  | PI2 | Monitoring & Intelligence  (Indicator 35) | 53.8 | The government should optimise usage of the existing system (e.g. collating and analysing the National Physical Fitness Standard - SEGAK data for children aged 10-17 years old) by ensuring appropriate feedback to parents and school management, strengthen referral mechanism for identified cases to the nearest health clinic as well as provide the follow-up of these cases. | 265 (3.5) | 2 | 7* |
|  | PI3 | Funding & Resources  (Indicator 39) | 40.4 | The government should substantially increase funding specific for population nutrition promotion so that it is commensurate with size of the population health burden that unhealthy diet creates. | 263 (3.4) | 3 | 9 |
|  | PI4 | Governance  (Indicator 31) | 42.7 | The government should continuously strengthen access to information related to public consultation (e.g. advocate and improve 'seranta awam' website to be more user friendly, interactive and open access for submissions by the main affected parties (e.g. non-governmental organisations, academia/ professional/ public and industry). | 250 (3.3) | 4 | 14 |
|  | PI5 | Funding & Resources  (Indicator 41) | 35.8 | The government should strengthen the sustainable funding and functioning of MySihat so that it becomes a significant force for health promotion (similar to ThaiHealth and VicHealth). | 249 (3.2) | 5 | 15 |
|  | PI6 | Monitoring & Intelligence  (Indicator 37) | 50.8 | The government should ensure sufficient resources (at least 5-10% of programme funding) and capacity building on evaluation of major programmes and policies related to nutrition and health plans. | 240 (3.1) | 6 | 16 |
|  | PI7 | Governance  (Indicator 31) | 42.7 | The government should continuously strengthen transparency of policy development (e.g. fully implementing the Guideline on Public Consultation Procedures). | 230 (3.0) | 7 | 19 |
|  | PI8 | Governance  (Indicator 30) | 43.8 | The government should continuously strengthen and capacity building on Regulatory Impact Statement (RIS) preparation by focusing on evidence-based approaches derived from public health perspective for policies which carry health implications. | 227 (3.0) | 8 | 21 |
|  | PI9 | Health-in-all Policies (Indicator 47) | 37.3 | The government ensures that health (broadly defined to encompass obesity and diet-related NCDs) impacts are taken into account in non-food policies using the existing platforms (e.g. Technical Working Group of Nutrition Policy), especially within urban planning and land zoning policies. | 226 (2.9) | 9 | 22 |
|  | PI10 | Health-in-all Policies (Indicator 46) | 45.0 | The government ensures that nutrition impacts are taken into account through Ministry of Agriculture and Agro-Based Industry (MOA) focusing on production of basic food supply based on population needs and demand, as well as relevant Ministries supporting the food and nutrition-related policies by Ministry of Health. | 216 (2.8) | 10 | 28 |
|  | PI11 | Governance  (Indicator 29) | 36.9 | The government should continuously strengthen conflicts of interest management for food industry engagement with policy development (e.g. instituting a lobby register) and among government officials (e.g. enacting the Political Donations and Expenditure Act and enforcing asset declarations for all staff). | 200 (2.6) | 11 | 29 |
| Third Pillar – "Prioritise Further Investigation" (PFI) | PFI1 | Food Retail  (Indicator 18) | 35.8 | The government should investigate the restrictions on the opening hours of fast food restaurants (e.g. amendment on the Licensing of Food Establishment By-Laws) and seek for opportunities to restrict the placement of new fast food outlets around schools and in residential areas. | 265 (3.5) | 1 | 7* |
|  | PFI2 | Food Promotion (Indicator 8) | 35.8 | The government should investigate policy options (e.g. regulation) to restrict non-broadcast marketing of unhealthy food and beverages to children. | 261 (3.4) | 2 | 10 |
|  | PFI3 | Food Composition (Indicator 1) | 43.1 | The government should investigate food composition standards in selected food groups for added sugar and saturated fats. | 259 (3.4) | 3 | 11 |
|  | PFI4 | Food Prices  (Indicator 10) | 46.9 | The government should investigate the price rises in fruit and vegetables and identify potential fiscal policies to address this increment. | 253 (3.3) | 4 | 13 |
|  | PFI5 | Food Provision (Indicators 14-17) | 53.8 | The government should measure the degree of implementation and reach of its various policies and programmes to support the provision of healthy food in ECEs/schools and other public and private sector organisations. | 236 (3.1) | 5 | 17 |
|  | PFI6 | Food Composition (Indicator 2) | 34.2 | The government should conduct situational analyses of the top '10' popular out-of-home meals (which includes *mamak*, hawker stands, fast food outlets etc.) and collect food samples, food composition analyses and recipe construction to identify key ingredients relating to total fat, sugar and salt in order to influence the composition of foods towards healthier profiles. | 226 (2.9) | 6 | 23 |
|  | PFI7 | Food Prices (Indicators 12-13) | 54.8 | The government should investigate policy options to provide healthy foods (e.g. food coupon as part of *Bantuan Rakyat 1Malaysia* (BR1M) for fresh fruits and vegetables, high fibre foods such as wholegrain products, low fat, sugar and sodium foods) to vulnerable groups including urban poor. | 223 (2.9) | 7 | 25 |
|  | PFI8 | Food Trade & Investments  (Indicator 23) | 43.5 | The government should investigate the opportunities to ensure that the provisions are in place in trade investment analysis (TIAs) to protect the policy space for food and nutrition-related policies. | 197 (2.6) | 8 | 30 |
| Fourth Pillar – "Prioritise Conditions for Planned Policies" (PCPP) | PCPP1 | Food Labelling (Indicator 3) | 61.2 | The government should implement planned regulations on mandatory nutrition labelling (sodium and total sugar) and quantitative ingredient declarations, as well as to broaden the declaration to include added sugars in the nutrient label. | 279 (3.6) | 1 | 2* |
|  | PCPP2 | Monitoring & Intelligence  (Indicators 33-34, 36, 38) | 57.3 | The government should continuously maintain and expand its programme of monitoring food environments and population nutrition with particular attention to representing vulnerable groups including the urban poor. | 232 (3.0) | 2 | 18 |
|  | PCPP3 | Platforms for Interaction  (Indicators 42-45) | 54.6 | The government should continuously ensure that there are robust mechanisms for collaborative engagements to reduce obesity and diet-related NCDs across government sectors and with the commercial sector, NGOs, academia, and communities. | 229 (3.0) | 3 | 20 |
|  | PCPP4 | Leadership  (Indicators 24-28) | 60.5 | The government should implement planned food policies, announced funding for nutrition programmes, and nutrition targets (in particular to the vulnerable groups including urban poor). | 220 (2.9) | 4 | 26 |

**Abbreviations:**

PP=Prioritise Policy (first pillar); PI=Prioritise Infrastructure (second pillar); PFI=Prioritise Further Investigation (third pillar); PCPP=Prioritise Conditions for Planned Policies (fourth pillar).
